# Qualitative Risk Assessment for Antimicrobial Resistance among Humans from Salmon Fillet Consumption Due to the High Use of Antibiotics against Bacterial Infections in Farmed Salmon

**DOI:** 10.3390/antibiotics11050662

**Published:** 2022-05-15

**Authors:** Marília Salgado-Caxito, Natalia Zimin-Veselkoff, Aiko D. Adell, Jorge Olivares-Pacheco, Fernando O. Mardones

**Affiliations:** 1 Escuela de Medicina Veterinaria, Facultad de Agronomía e Ingeniería Forestal, Facultad de Ciencias Biológicas y Facultad de Medicina, Pontificia Universidad Católica de Chile, Santiago 7820244, Chile; mariliasalgadocaxito@gmail.com (M.S.-C.); natalia.zimin@uc.cl (N.Z.-V.); 2 Millennium Initiative for Collaborative Research on Bacterial Resistance (MICROB-R), Santiago 7550000, Chile; aiko.adell@unab.cl; 3 Escuela de Medicina Veterinaria, Facultad Ciencias de la Vida, Universidad Andrés Bello, Santiago 8320000, Chile; 4 Grupo de Resistencia Antimicrobiana en Bacterias Patógenas y Ambientales GRABPA, Instituto de Biología, Facultad de Ciencias, Pontificia Universidad Católica de Valparaíso, Valparaíso 2340025, Chile; 5 Department of Pediatric Infectious Diseases and Immunology, Facultad de Medicina, Pontificia Universidad Católica de Chile, Santiago 8331150, Chile

**Keywords:** aquaculture, Chile, food animal production, food safety, qualitative risk analysis, salmon farming

## Abstract

**Background:** Worldwide, aquaculture is considered as a hotspot environment for antimicrobial resistance (AMR) due to the intense use of antibiotics in its productive systems. Chile is the second largest producer of farmed salmon worldwide, and tons of antibiotics are used to control bacterial diseases, such as Salmon Rickettsial Syndrome (SRS) and Bacterial Kidney Disease (BKD). However, studies determining the risk of consuming salmon fillets that have been treated with antibiotics during the salmon production are limited. Consulting leading experts in the field could provide a knowledge base to identify and address this question and research gaps. **Methods:** Multisectoral risk perception of AMR through salmon fillet consumption was evaluated by eliciting expert data obtained through discussions during a workshop and from questionnaires given to experts from academia (n = 15, 63%), the public sector (n = 5, 21%), and the salmon industry (n = 4, 17%). **Results:** The qualitative risk analysis suggested an overall ‘low’ probability of AMR acquisition by consumption of salmon fillet that had been treated during the production cycle. The risk perception varied slightly between production stages in freshwater and seawater. In consensus with all sectors, this overall ‘low’, but existing, risk was probably associated with bacterial infections and the use of antibiotics. **Conclusions:** As it is essential to reduce the use of antibiotics in the Chilean salmon industry, this intersectoral approach and consensual results could favor effective implementation of targeted initiatives for the control and prevention of major bacterial diseases.

## 1. Introduction 

Aquaculture is a growing productive activity worldwide, providing sources of food and economic income for the population of many countries [1,2]. One of the most important sectors of aquaculture in Chile is the production of farmed salmon [3]. With more than 1000 tons of salmon biomass harvested, the country is considered the second largest salmon producer in the world [4]. However, the sustainability of its national industry has been affected by the high use of antibiotics to control relevant bacterial infections, such as *Piscirickettsia salmonis* (Salmon Rickettsial Syndrome or SRS) and *Renibacterium salmoninarum* (Bacterial Kidney Disease or BKD) [5,6]. Despite the reduction in their use for almost a decade, Chile still shows the highest consumption rates of the use of antimicrobials per ton of salmon produced in the world with approximately 380 tons of antibiotics used in 2020 [4]. This intense use of antibiotics, especially florfenicol and oxytetracyclines, could result in selecting antibiotic-resistant-bacteria (ARB) and/or antibiotic-resistance genes (ARGs) [7]. 

The mitigation of the antimicrobial resistance (AMR) phenomenon has been considered a priority by the World Health Organization (WHO), the Food and Agriculture Organization of the United Nations (FAO), and the World Organization for Animal Health (OIE). These institutions have established a tripartite intersectoral collaboration (https://www.fao.org/publications/card/en/c/CA2942EN, accessed on 11 May 2022) to address key points to tackle AMR at the human–animal-environment interface, which includes the extensive use of antibiotics in animal production systems [8]. Chile has also adopted various national regulations to control and reduce the use of antimicrobials. A good example is the implementation of electronic prescriptions for aquatic animals (issued exclusively by veterinarians), allowing real-time surveillance as part of the “National Plan to Combat Antimicrobial Resistance” launched in 2017 by the Ministry of Health [9]. In addition, multiple sanitary control procedures are established in the “Manual of Good Practices in the Use of Antimicrobials and Antiparasitics in Chilean Salmon Farming” that salmon producers must follow [10], and there are also initiatives coordinated by non-governmental organizations, such as the “Chilean Salmon Antibiotic Reduction Program (CSARP, https://www.csarp.cl/, accessed on 11 May 2022)”. However, diseases such as SRS and BKD are difficult to control and have resulted in a massive use of florfenicol and oxytetracycline in farmed salmon [4,5], increasing the risks of AMR.

Limited research has been carried out to study the impact of the consumption of antibiotic-treated salmon fillet on public health [7,11]. Previous studies have reported that the use of antimicrobials in animals has been positively associated with the likely emergence, maintenance, and spread of ARB and ARGs [12,13,14]. Other studies suggest that food derived from treated animals would more likely harbor ARB and ARGs, posing a risk of transmission to consumers, especially when eating raw (e.g., seafood) or undercooked meats [7,15,16]. Despite this, it remains unclear whether reduction in the use of antibiotics in food animals could directly lower the risk of AMR acquisition by humans (especially those consuming animal-origin food).

Consulting the opinion of professionals with proven expertise in a particular field of interest has been widely used when there are insufficient baseline data for immediate interventions with informed decision making, as well as promoting communication and awareness of the need to study a specific topic [6,17,18,19]. Through a systematic process of information gathering, evaluation, and documentation, a qualitative or quantitative risk assessment enables the assignment of a level of probability of occurrence for a specific event until more accurate information is available [7,20,21,22,23]. The aim of this study was to determine the multisectoral risk perception of AMR through salmon fillet consumption and to provide estimates of risk levels in each step of the salmon production cycle, using a structured expert elicitation process based on validated methodologies [21,24]. In addition, this expert elicitation highlighted production issues that could impact the Chilean farmed salmon trade and markets and, consequently, the sustainability of the national industry. This risk assessment could help decision makers in salmon farming systems to identify the most necessary steps for interventions to reduce the amount of antibiotics needed, achieve economic sustainability, and reduce AMR in aquatic animal production.

## 2. Methods and Materials

### 2.1. Process for the Identification and Selection of Experts

Experts from the salmon industry and the public sector were identified based on previous research focused on sanitary and production aspects [5,6,19]. Academic experts were identified based on their record of scientific publications relevant to the topics (i.e., AMR and/or salmon diseases). A total of 24 national and international (i.e., Brazil and Portugal) experts from academia (n = 15, 62.5%), the public sector (n = 5, 20.8%), and the salmon industry (n = 4, 16.7%) agreed to participate in a workshop called *“Brainstorming and expert workshop: Risk assessment for antimicrobial resistance from farmed salmon in Chile–A preliminary qualitative risk analysis”*. This workshop was facilitated by Pontificia Universidad Católica de Chile on 12 June 2019 to highlight and rate the main events that could represent a risk for emergence and dissemination of AMR during the production chain of salmon farming. Considering the interaction that salmon farms can have with natural environments, a participant from the Chilean non-governmental organization Melimoyu Ecosystem Research Institute (MERI) was also invited. MERI is responsible for promoting scientific research for the conservation of ecosystems in northern Chilean Patagonia and operates mainly in the region where most of the salmon farms are located.

For the selection of experts, the following inclusion criteria were considered, depending on the sector to which the expert belonged:

(1) Academia: researchers who have at least two peer-reviewed papers in the areas of antibiotic resistance, salmon pathogens, epidemiology of aquaculture production, and fish immunology.
(2) Public sector: professionals (veterinarians, aquaculture engineers, and marine biologists) working on public and/or animal health in governmental organizations, including the National Fisheries and Aquaculture Service (Servicio Nacional de Pesca y Acuicultura; SERNAPESA); Subsecretary of Fisheries and Aquaculture (Subsecretaria de Pesca y Acuicultura; SUBPESCA); Fisheries Development Institute (Instituto de Fomento Pesquero; IFOP); Agricultural and Livestock Service (Servicio Agrícola Ganadero; SAG); Chilean Food Safety Agency (Agencia Chilena Para la Inocuidad Alimentaria; ACHIPIA); and the Institute of Public Health (Instituto de Salud Pública; ISP).
(3) Salmon industry: members of the Salmon Technological Institute (INTENSAL, an organization for scientific and technical support to the production and supply companies of the entire national salmon industry composed of national and international professionals from different areas of the public and private sectors); veterinarians, biochemists, and biologists associated with diagnostic laboratories for aquaculture production in Chile.

### 2.2. Discussion for Identification of Nodes and Expert Elicitation

Based on the OIE recommendations [24], risk was defined as the exposure to specific hazards resulting in bacterial infections that do not respond to antibiotic therapies and that have arisen from the consumption of farmed salmon, consumption of their subproducts, or exposure to salmon farm settings. Experts used this risk definition to conduct the expert elicitation process. The process of the qualitative risk assessment combined the characterization of three main components: (i) identification of the hazards (pathogenic agents that cause the event); (ii) evaluation of the exposure levels of an individual or population to these potential hazards; and (iii) appraisal of the context (socio-economic, ecological, or cultural) in which the event may occur [7].

First, participants identified units of interest corresponding to the events that could be analyzed. The interactive audience engagement platform, Mentimeter (https://www.mentimeter.com, Mentimeter AB public limited company, Stockholm, Sweden, accessed on 12 June 2019), was used during the development of the workshop [25]. This software was used to conduct online surveys in real time using different types of questions (i.e., word cloud, ratings, multiple choice), allowing us to obtain broad expert opinions as well as to encourage discussion of the different points addressed. Through these discussions, the participants were able to gather information on the state of the art of AMR and the use of antibiotics in aquaculture worldwide and draw upon their own expertise to define the events that could be related to emergence of ARB and ARGs among humans due to the consumption of treated salmon fillet in Chile. From here on, these events will be referred to as nodes.

After the identification of the nodes and the three main components needed for the qualitative risk assessment (hazards, exposure levels, and the context in which the event may occur), a second data capture was carried out to reach consensus on which would be the potential risk pathways and the estimated level of risk for each among the steps or situations that could be faced within the production cycle (defined as scenario in this manuscript). To accomplish this, experts were individually surveyed to provide their best estimates of the risk for the major pathways within salmon farming, along with the associated level of uncertainty to achieve a high level of confidence in the results. Questionnaires included 53 questions that were clustered into four sections: (i) freshwater phase; (ii) seawater phase; (iii) salmon processing chain; and (iv) impact of farmed salmon consumption on human health. The questions are available in English and Spanish as Supplementary Material Table S1.

#### 2.2.1. Identification of the Hazards of AMR in Salmon Farming

According to the OIE’s *Aquatic Animal Health Code* guideline, hazard in AMR risk analysis is defined as a resistant microorganism or the resistance determinants that emerge because of the use of a specific antimicrobial agent in fish [7,24]. This definition reflects the potential for resistant microorganisms to cause adverse health effects, as well as the potential for horizontal transfer of AMR genetic determinants between microorganisms. In the current study, hazards included any ARB representing a threat to human health that could be selected or co-selected using oxytetracycline or florfenicol (as well as the ARGs that promote this antibiotic-resistance phenotype) and with high potential of transmission.

#### 2.2.2. Evaluation of the Levels of Exposure of an Individual or Population to These Potential Hazards

The exposure characterization was performed considering the potential sources of the hazards from which AMR could emerge (i.e., human, animal, and environmental), the possible routes of transmission within and between sources (risk pathways), and the biological determinants or environmental elements that could play a role in the emergence and transmission of ARB and ARGs. 

Although there is still a significant lack of knowledge about the biological determinants and environmental elements related to the prevalence and spread of AMR in aquatic environments or the impact that anthropogenic sources could have on these issues [26,27], 10 factors that could promote the emergence, acquisition, mobilization, and maintenance of AMR in aquaculture were presented to the experts to assist them in their evaluation, including:

(1) Frequency of oral or medicated feed administration of oxytetracycline and/or florfenicol to treat specific bacterial infections of farmed salmon.
(2) Proportion of commensal bacteria that are capable of developing resistance along with the mechanisms and pathways of direct and indirect transfer of ARGs to human pathogenic bacteria.
(3) Human infections caused by oxytetracycline and/or florfenicol resistant bacteria acquired by people through salmon consumption.
(4) The pharmacokinetic and pharmacodynamic aspects of these antibiotics.
(5) Number of fish treated with antibiotics in Chile.
(6) The possibility of using antibiotics without a veterinarian prescription.
(7) The probability of co-selection, cross-resistance (a single antibiotic resistance mechanism conferring resistance to more than one antibiotic), or co-resistance (multiple antibiotic resistance mechanisms) with other antibiotic classes.
(8) The decrease in antibiotic usage in Chile in the last decade [4] associated with the improvement in farming practices and better vaccines.
(9) The potential link with virulence genes and ARGs.
(10) Surveillance data on fish pathogens and trends of ARB and ARGs in animals, products and subproducts of animal origin, or livestock wastes.

The above factors were presented as a guide to provide experts with a better understanding of potential drivers for emergence and spread of AMR in aquatic animal production. In this regard, they were instructed to take these factors into consideration along with their previous experience when evaluating the levels of exposure to AMR microorganisms that a human might face when eating antibiotic-treated salmon fillet.

#### 2.2.3. Appraisal of the Context

The factors that can promote the emergence and dispersion of ARB and ARGs in salmon farms in Chile were evaluated at the policy level (regulations and guidelines to control AMR), at the systems level (policy implementation, health system management, wastewater management, and agriculture and livestock management), and at the individual level (human behavior and cultural factors). 

### 2.3. Risk Assessment

Experts were asked to evaluate the scenarios of the salmon production cycle and provide a qualitative analysis of the risk of emergence and spread of ARB and/or ARGs among humans associated with salmon consumption. For this, they rated the probability of an identified node (or more) to be related in some way to each pathway as ‘insignificant’ (the event occurs in exceptional circumstances), ‘low’ (the event occurs sporadically), ‘moderate’ (the event occurs regularly), or ‘high’ (the event occurs in most circumstances). Each step of this evaluation was considered as formal data acquisition, and participants’ elucidation was our main data source, complemented by secondary databases (i.e., literature review and a previous study on animal health professionals’ KAP on AMR in aquaculture that was not published). 

To facilitate analysis, qualitative estimation was converted to quantitative data using a risk ordinal scale ranging from 0 to 3 adapted from another study [17]. Briefly, numerical values were assigned to each qualitative estimation, where ‘0’ corresponded to the lowest possibility of risk (insignificant), ‘1’ to low risk, ‘2’ to moderate risk, and ‘3’ to the highest risk. Although respondents answered according to their area of expertise (and, therefore, questionnaires did not have the same response rate), all experts were considered to have equivalent levels of knowledge and estimates were weighted equally. Usually, the risk assessment process is based on the principle of combining the level of the likelihood and the degree of the consequence for the risk event to occur [7]. This is because events (in this manuscript referred to as nodes) with the same probability can have outcomes ranging from minor to more serious, resulting in the need to model risk in terms of both probability and outcome. However, in the current study, a single consequence was associated with each probability of occurrence of a hazardous event using an assumption that risk represented the simple (unweighted) product of the degree of the probability (from 0 to 3) and of the degree of the consequence (in this case = 1, meaning the onset of AMR among consumers of salmon fillet treated with antibiotics was a single event).

Another factor taken into consideration was whether the evaluated nodes were conditional (when an event is assumed to be totally conditional on the previous event) or conditional-independent. Thus, a risk matrix was used in which probabilities were combined following a methodology described previously [28]. Therefore, conditional nodes had their probabilities sequentially multiplied for the final probability estimate (Table 1), considering that the result had to be equal to or less than the smallest probability [28,29]. To our knowledge, there are no specific matrices for combining qualitative independent probabilities. Therefore, a general probability of conditional-independent nodes was obtained by summing their probabilities using the matrix published by Moutou et al. [30] (Table 2). Individual responses were included in a spreadsheet obtaining the median score from all experts at each node. Median scores were used in the combination matrices for either conditional or conditional-independent risk estimates. All analyses were carried out using an Excel spreadsheet. The nodes, scenarios, and risk values assigned to each pathway are described in the Results Section.

## 3. Results

During the expert workshop, a flowchart was collaboratively constructed in which nine nodes (i.e., events) were identified by combining opinions from five independent groups of experts. Each node corresponds to an event that could be related in some way to human acquisition of ARB and/or ARGs from consumption of salmon fillet treated with florfenicol or oxytetracycline (Table 3). 

**Figure 1:**
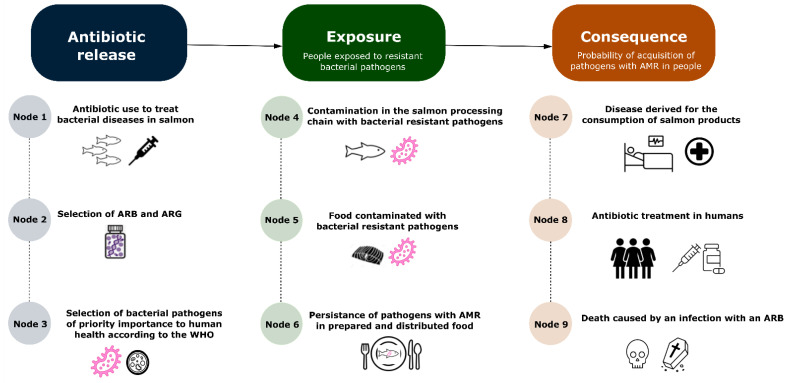
Risk nodes (event under analysis) for emergence and spread of antimicrobial resistance (AMR) among humans through consumption of farmed salmon leading to serious consequences (e.g., death) identified by the experts that participated in the workshop *“Brainstorming and expert workshop: Risk assessment for antimicrobial resistance from farmed salmon in Chile–A preliminary qualitative risk analysis”*. ARB: antibiotic-resistant bacteria; ARG: antibiotic resistance gene; WHO: World Health Organization.

Nodes were clustered and then classified into three groups: ‘Antibiotic Release’, ‘Exposure’, and ‘Consequence’ (Figure 1). The events that could have causal or influential relationships are connected by dotted lines, while the direction of sequential events is indicated by arrows. The lack of connectors represents conditional independence since the probability of a specific node may (or may not) be affected by previous ones. For example, the occurrence of one or more nodes within ‘Antibiotic Release’ could increase the probability of nodes within the ‘Exposure’ group, although these could also occur independently of nodes 1, 2, or 3.

**Figure 2:**
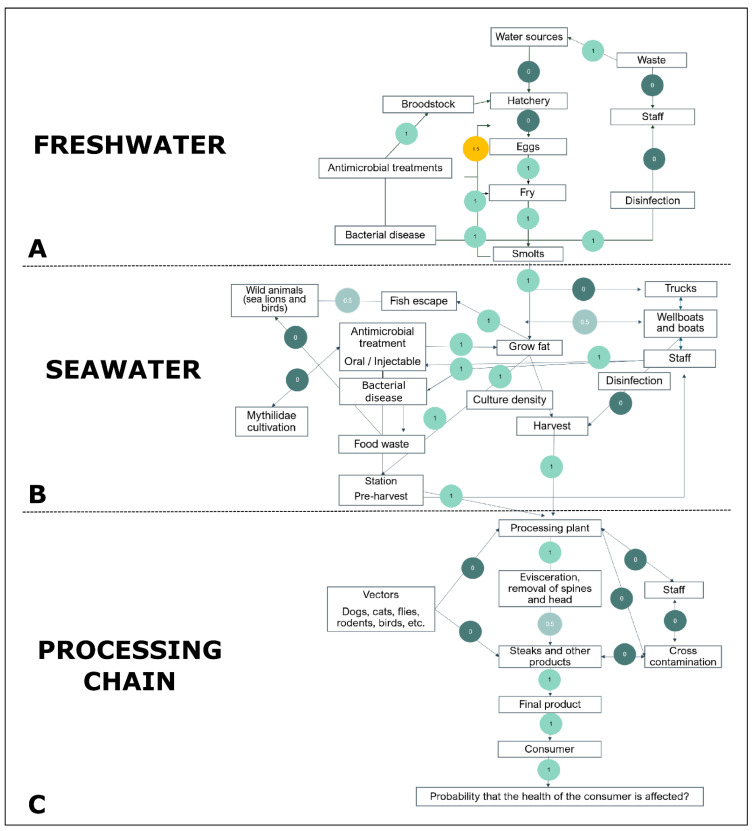
Rectangles represent the scenarios throughout the salmon production. Solid lines represent causal or influential relationships between scenarios. Arrows indicate the pathways (possible uni- or bidirectional routes of transmission) within the salmon production. The colored circles represent the weighted risk value for AMR through salmon consumption (from 0 to 3) assigned to each pathway that was calculated using the results obtained from questionnaires applied to experts from academia, the public sector, and the salmon industry and from the expert elicitation in the workshop *“Brainstorming and expert workshop: Risk assessment for antimicrobial resistance from farmed salmon in Chile: a preliminary qualitative risk analysis”*. (**A**) Scenarios and risk values attributed to the pathways during freshwater salmon farming. (**B**) Scenarios and risk values attributed to the pathways during seawater salmon farming. (**C**) Scenarios and risk values attributed to the pathways in the processing chain of salmon farming.

The results of the expert elicitation obtained through the discussions during the workshop, including the identification of the nodes, and from the questionnaires answered by each expert individually, were used to generate a diagram of the salmon production within the farm settings (i.e., freshwater phase, seawater phase, and processing chain) (Figure 2). 

A total of 34 scenarios were identified throughout salmon farming, including 11 from the freshwater phase (Figure 2A), 14 from seawater (Figure 2B), and 9 from the processing chain (Figure 2C). A brief description of the scenarios analyzed, corresponding to steps or situations within the salmon production, is provided in Table 4.

In the created diagram (Figure 2), scenarios were represented by rectangles, and those that were considered to have a causal or influential relationship with each other were connected by solid lines. It was possible to obtain the multisectoral risk perception of 38 pathways among scenarios by eliciting expert data. These (uni- or bidirectional) routes of transmission within the salmon production are indicated in the diagram by arrows, including 12 in the freshwater phase (Figure 2A), 15 in the seawater phase (Figure 2B), and 11 in the processing chain (Figure 2C). 

Overall, there was a consensus between academia, public sector, and salmon industry experts that the risk of acquiring ARB and ARGs from consumption of treated salmon might be ‘low’, and most pathways identified as threats during the production of farmed salmon were ranked with a risk level between ‘insignificant’ and ‘low’ (nodes might occur under exceptional circumstances or sporadically, respectively). However, experts broadly agreed that risks were most likely associated with bacterial infections and antibiotic therapies. The risk analysis showed a perception that the freshwater phase of salmon production would have a proportionally larger number of pathways associated with at least a ‘low’ level of risk (67%, 8/12, Figure 2A) compared to the seawater phase (60%, 9/15, Figure 2B) and the processing chain (36%, 4/11, Figure 2C).

Most pathways with an associated risk in the freshwater production were preceded by the ‘Antimicrobial treatments’ scenario towards the ‘Broodstock’, ‘Eggs’, and ‘Fry’ scenarios (Figure 2A). The pathway between the scenarios ‘Antimicrobial treatments’ and ‘Eggs’ had the highest level of risk assigned (‘moderate’) as ‘Eggs’ is one of the scenarios in which most treatments are carried out. In addition, a ‘cyclical risk’ of maintenance of ARB, ARGs, or antibiotic residues among the scenarios ‘Eggs’, ‘Fry’, and ‘Smolts’ was identified by the experts due to the possibility of water reuse and/or poor cage cleaning. Bacterial diseases were also included in the flowchart, not only because of their causal relationship to the need for antibiotic therapies, but also because they result in a risk of ARB/ARG emergence from the increased use of antibacterial products needed for disinfection. A ‘low’ risk was also perceived in releasing waste in the water sources used in this phase due to the possibility of absence of water treatment before use by salmon farms.

Like the freshwater phase, many risk pathways in the seawater phase of the salmon production were related to antimicrobial usage. ‘Grow fat’ was the scenario with the highest number of perceived risk pathways, both toward the earlier and later scenarios (Figure 2B). The staff were also perceived to pose a risk for the emergence and spread of ARB and ARGs that was mainly associated with the handling of farmed salmon infected with bacterial diseases and the administration of antibiotics to these animals. Additionally, a perception of low risk was linked by some experts to AMR pollution from anthropogenic sources (i.e., wellboats, boats, and trucks) and to the influential relationship within the environment (between wild animals and escaped salmon). However, when gathering all qualitative estimations and converting to our ordinal-scale ranking (from 0 to 3), the risk in the pathways associated with both scenarios was quantified as less than 1, and it was considered as ‘insignificant’. 

Concerning the processing chain, in all internal pathways some degree of risk was identified (Figure 2C). Nonetheless, the risk of the pathway after the ‘Evisceration and removal of bones and head’ scenario was rated as ‘insignificant’ (less than 1 on our ordinal scale) and the remaining ones were considered as ‘low’. On the other hand, as opposed to the results obtained for the seawater phase, no external factors (i.e., environmental sources) nor anthropogenic contamination were associated with an AMR risk.

## 4. Discussion

The high use of florfenicol and oxytetracycline during salmon farming to control bacterial diseases (e.g., SRS and BKD) could represent a public health concern. However, it is unclear whether the consumption of farmed salmon treated with antibiotics may be a determining factor for the acquisition of ARB and/or ARGs by the human population. In the absence of scientific evidence, this risk was explored by 24 experts of different areas that evaluated the main scenarios and pathways within salmon production as a first approach to generate baseline knowledge and encourage studies in the field. There was a perception of ‘low’ risk of ARB and ARGs among the population through salmon consumption. However, most of the pathways assigned with some degree of risk were those preceded by the use of antimicrobials, highlighting that the control of bacterial diseases requires special attention. 

The presence of professionals from academia, the public sector, and the salmon industry sectors provided a comprehensive and holistic understanding of the risks of AMR from the consumption of salmon fillet. As this is a multidisciplinary problem, professionals from the salmon industry were included to capture the perception within the national salmon farming routine. Importantly, none of the salmon industry participants were farmers or directly involved in salmon production, but service providers for the salmon farms (i.e., scientific, technical, and/or diagnostic support). All experts had a proven scientific background and formed a well-balanced panel of experts, which allowed for the inclusion of different perspectives, as recommended by other authors [21]. 

A higher proportion of risk pathways (perceived as ‘low’ or ‘moderate’ risk) was identified in the freshwater phase (Figure 2A). However, most diagnostics and preventive measures are mainly carried out in the seawater phase, probably because of the low economic impact bacterial diseases have in the freshwater phase [31,32,33]. The use of antibiotics in the freshwater phase is common and may represent an important risk factor for the emergence of AMR [7,12,13,34,35]. In fact, ‘Eggs’ was the scenario with the highest assigned risk (‘moderate’) (Figure 2A) due to the high frequency of individual antibiotic treatments on all animals in the cage. Another concern is about the available data from the freshwater phase. While electronic prescriptions are a good source of information on amounts of antibiotics used in this phase, other relevant data are not adequately recorded (e.g., frequency of detection of bacterial AMR infections, number of unresponsive antibiotic treatments) [32,33]. Insufficient efforts to diagnose and prevent bacterial disease, high frequency of antibiotic use, and limited data recording demonstrate that the freshwater phase should be a priority for intervention to improve data recording and to implement initiatives seeking to reduce antibiotic use.

Risks were clearly associated with bacterial infections and the consequent use of oxytetracycline and florfenicol. Good sanitary practices are essential for reducing infectious diseases [36,37]. However, strategies should go beyond the use of antibacterial products that also contribute to the selection of resistant pathogens [38,39]. Promoting proper disposal of organic matter, hand washing when handling sick animals, ensuring good animal nutrition, reducing animal stress, and avoiding overcrowding could help prevent and control bacterial infections. The reduction in antibiotic treatments could also be reflected in less aquatic environmental contamination, thus decreasing public health risks. It is estimated that 75% of antibiotics fed to fish are not metabolized, exacerbating this contamination if waters are not treated before being returned to rivers [7]. In addition, antibiotic administration in salmon farming is mainly carried out through medicated feeds, which have lower palatability and may favor the spread of antimicrobial residues through degraded feed [5,33,40,41,42,43,44]. To our knowledge, there is no surveillance or monitoring of the water sources used on salmon farms, which could be affecting other food production systems intended for human consumption that are supplied by the same rivers, such as livestock and agriculture irrigation.

To our knowledge, this is the first study in Latin America to review the entire salmon production chain and the potential human health risks associated with the emergence and spread of ARB and ARGs through the consumption of farmed salmon. Despite the intense use of antibiotics during the salmon production in Chile, the risk of occurrence and spread of ARB and ARGs associated with salmon consumption was understood as ‘low’. National initiatives, such as the prohibition of prophylactic treatments, mandatory diagnosis before treating a bacterial disease, and the surveillance program for oxytetracycline and florfenicol resistance in *P. salmonis* (SRS) [9,10,33,45], could be some reasons for experts to consider the risk of AMR by salmon consumption to be low. The data obtained up to 2020 have shown no resistant isolates of *P. salmonis* to oxytetracycline or florfenicol. However, 90% of the isolates obtained in 2019 presented a reduced susceptibility to florfenicol and 70% to oxytetracycline [33,46]. This corroborates the experts’ conclusions, since it shows that the risk exists but, as it is a slow phenomenon, the risk is therefore very low. The implementation of the antimicrobial surveillance program for *R. salmoninarum* (BKD) and *Flavobacterium psychrophilum* in 2020 should provide more information on the trends of the AMR phenomenon in the main bacterial pathogens of farmed salmon in Chile. 

Although there is evidence that this low risk is realistic and representative of the current situation, the possibility of underestimation cannot be ruled out. Moreover, the experts contributed their knowledge according to their field, and the estimates were not weighted. Therefore, the questionnaires do not have the same response rate, and a possible survey response bias could not be quantified. While rapid risk assessment is useful in identifying the phases and pathways requiring special attention, it can only be considered as preliminary in the comprehensive risk assessment. One way to complement this type of analysis and obtain a more reliable result is to use a One Health approach integrating surveillance focused on organisms that may pose a threat to human health or have the potential to acquire ARGs and develop antibiotic resistance (e.g., *E. coli*) [7]. Integrated surveillance, which consists of simultaneously investigating AMR in livestock, humans (i.e., patients), and the food production chain using systematized methodology [47,48,49], could help us to quickly assess the consequences to human health arising from different sources. Given its multifactorial characteristics, One Health AMR surveillance could enable early detection of emerging resistance threats and ensure comprehensive data capture for implementation of specific mitigation strategies and AMR risk trends assessment for consumers. Finally, the results of this study yielded the identification of critical points in the salmon production system and areas where research and data collection are necessary to reduce the uncertainty of risk estimates identified in this study. Thus, the same methodology could be used to evaluate other animal production systems that also show high rates of antibiotic use and could represent a public health risk. 

## 5. Conclusions 

To the best of our knowledge, this qualitative risk assessment is the first overview of potential risks of AMR for humans from the consumption of salmon fillet by consulting experts from different fields related to public and animal health. Although the possibility of underestimation cannot be excluded, there was a consensus among these sectors on the low, but existing, risk of AMR through salmon consumption. It should also be noted that a possible survey response bias could not be quantified and that this estimate should be considered as preliminary in the comprehensive risk assessment. In addition, this risk was mainly linked to the use of oxytetracycline and florfenicol, particularly in the freshwater phase. Improved data recording and implementation of enhanced preventive measures to mitigate bacterial diseases and reduce antibiotic use at this phase could promote economic sustainability and food safety in relation to AMR from antibiotic-treated salmon consumption. Furthermore, such initiatives could be facilitated by the observed intersectoral alignment. The expert elicitation exposed in this study generates a baseline information of the AMR risks resulting from the consumption of salmon treated with antibiotics and highlights the need for further studies, especially in the steps identified by the experts as priorities. Finally, the present study could be replicated in other countries and/or for other animal productions looking for strategies to curb AMR, especially in scenarios where high amounts of antibiotics are used. A comprehensive strategy using the “One Health” approach is needed to mitigate potential antibiotic resistance in animal production by considering key factors, such as bacterial disease prevalence, antibiotic use patterns, and human–animal interactions.

## Figures and Tables

**Table 1:** Combination matrix generated to unify the conditional risk estimates, which include factors that assume that the second event (i.e., node) is totally conditional on the previous event (first event).

First Event	Second Event			
	Insignificant ^1^	Low ^2^	Moderate ^3^	High ^4^
Insignificant	Insignificant	Insignificant	Insignificant	Insignificant
Low	Insignificant	Low	Low	Low
Moderate	Insignificant	Low	Moderate	Moderate
High	Insignificant	Low	Moderate	High

^1^ ‘Insignificant’ refers to the events occurring under exceptional circumstances. ^2^ ‘Low’ refers to the events occurring sporadically. ^3^ ‘Moderate’ refers to the events occurring regularly. ^4^ ‘High’ refers to the events occurring in most circumstances.

**Table 2:** Combination matrix generated to unify the conditional-independent risk estimates, which include factors that assume that the second event (i.e., node) is independent of the previous event (first event), although it may be affected if the first event occurs.

First Event	Second Event			
	Insignificant ^1^	Low ^2^	Moderate ^3^	High ^4^
Insignificant	Insignificant	Low	Low	Moderate
Low	Low	Low	Moderate	Moderate
Moderate	Low	Moderate	Moderate	High
High	Moderate	Moderate	High	High

^1^ ‘Insignificant’ refers to the events occurring under exceptional circumstances. ^2^ ‘Low’ refers to the events occurring sporadically. ^3^ ‘Moderate’ refers to the events occurring regularly. ^4^ ‘High’ refers to the events occurring in most circumstances.

**Table 3:** Definition of nodes that could result in ARB and/or ARGs acquisition by humans through consumption of farmed salmon that has been treated with florfenicol or oxytetracycline during the salmon production cycle.

Node	Definition
Node 1	Probability of the need to use florfenicol or oxytetracycline to treat bacterial infections in farmed salmon.
Node 2	Probability of selection of ARB and ARGs given that the previous event has occurred.
Node 3	Probability that ARB and ARGs have been selected and that these are considered as a priority AMR pathogen for human health by the WHO.
Node 4	Probability that the processing chain has been contaminated with bacteria-resistant pathogens.
Node 5	Probability that salmon fillet has been contaminated with bacteria-resistant pathogens given that the previous event has occurred.
Node 6	Probability of persistence of AMR pathogens in the contaminated salmon fillet after preparation and distribution.
Node 7	Probability of adverse health effects caused by resistant microorganism or resistance determinants due to the consumption of salmon fillet treated with oxytetracycline or florfenicol.
Node 8	Probability of the need to use antibiotics to treat bacterial infections among consumers of salmon fillet treated with antibiotics given that the previous event has occurred.
Node 9	Probability of death caused by a bacterial infection that did not respond to antibiotic therapies and that has originated from the consumption of salmon fillet treated with florfenicol or oxytetracycline.

ARB: antibiotic-resistant bacteria; ARG: antibiotic resistance genes; WHO: World Health Organization.

**Table 4:** Brief description of the main scenarios within the salmon production cycle.

Phase of the Salmon Production	Scenario	Definition
Freshwater/Seawater	Antimicrobial treatments	The use of antibiotics to eliminate or inhibit the growth of bacteria in a living organism. The main antibiotics used in salmon farming are florfenicol and oxytetracycline.
Freshwater/Seawater	Bacterial diseases	Refers to the main bacterial diseases in farmed salmon, including Salmon Rickettsial Syndrome (caused by *Piscirickettsia salmonis*) and Bacterial Kidney Disease (caused by *Renibacterium salmoninarum*).
Freshwater/Seawater	Disinfection	A sanitary practice to prevent infectious diseases by using bactericidal or bacteriostatic products to eliminate or inhibit the growth of bacteria on inert surfaces.
Freshwater/Seawater/Processing chain	Staff	All professionals working in at least one stage within the salmon production either in the salmon farms or in the processing chain.
Freshwater	Broodstock	A group of mature salmon individuals used for breeding.
Freshwater	Eggs	Farmed salmon eggs recovered from breeding.
Freshwater	Fry	Farmed salmon fry, from the hatched eggs in alevin to the onset of smoltification.
Freshwater	Hatchery	Incubation period of farmed salmon eggs recovered from breeding.
Freshwater	Smolts	Period when juvenile farmed salmon initiate their physiological adaptation to live in a marine (saltwater) environment.
Freshwater	Waste	Refers to the liquid wastes produced by industry that could be released into aquatic environments.
Freshwater	Water sources	Rivers that supply salmon farms and, eventually, other production systems (i.e., livestock and agricultural irrigation) and communities.
Seawater	Culture density	Refers to the number of farmed salmon by the size of the cages in the seawater phase.
Seawater	Fish escape	Refers to farmed salmon that manage to escape from the pens into the local aquatic environment.
Seawater	Food waste	Refers to residues of medicated feed used for antibiotic administration in salmon farming.
Seawater	Grow fat	Rearing and fattening period of farmed salmon during the seawater phase.
Seawater	Harvest	Process in which salmon that finish the Grow fat step are extracted for human consumption.
Seawater	Mythilidae cultivation	Mytilidae farms that are located near the salmon farms.
Seawater	Pre-harvest station	An empty farm typically nearby a processing plant that receives from another farm market-size fish a few hours before harvest at the processing plant. Sometimes called a collection center.
Seawater	Trucks	Refers to the trucks used to transport farmed salmon.
Seawater	Wellboats and boats	Refers to the wellboats (a fishing vessel) used for storing and transporting live farmed salmon and the boats used to visit the salmon farm pens.
Seawater	Wild animals	Refers to wild animals that can be found in (or near) the aquatic environments of salmon farms.
Processing chain	Consumer	The person who purchased and/or consumed farmed salmon.
Processing chain	Cross-contamination	Refers to the process by which food comes into contact with external substances, generally harmful to health.
Processing chain	Evisceration, removal of spines and head	Refers to the removal of the viscera (i.e., intestines) and inedible parts of a salmon carcass for the preparation of the final product.
Processing chain	Final product	Refers to salmon fillets or their subproducts ready to be marketed.
Processing chain	Probability that the health of the consumer is affected	Refers to the final product contaminated with AMR pathogens available for sale and consumption.
Processing chain	Processing plant	A place where various operations are carried out to process, handle, and store harvested salmon for human consumption.
Processing chain	Steaks and other products	Refers to the preparation of final products by cutting the salmon meat into fillets or subproducts before packaging.
Processing chain	Vectors	Refers to any animal that can act as a carrier of ARB or ARGs and contaminate the processing chain.

## Data Availability

The data presented in this study are available on request from the corresponding author. The data are not publicly available due to ethical reasons as it was obtained non-anonymously from professionals through discussions and the risk analysis questionnaires.

## Supplementary Materials

The following supporting information can be downloaded at: https://www.mdpi.com/article/10.3390/antibiotics11050662/s1, Table S1: Questionnaire individually applied to experts from academia, the public sector, and the salmon industry to capture risk estimations for the major pathways within salmon farming.
